# Development and Preliminary Testing of the Staffordshire Questionnaire for Adolescent Idiopathic Scoliosis (SQ‐AIS): Content and Face Validity

**DOI:** 10.1002/hsr2.70213

**Published:** 2024-11-22

**Authors:** Enza Leone, Nachiappan Chockalingam, Robert Needham, Aoife Healy, Nicola Eddison, Nikola Jevtic, Vinay Jasani

**Affiliations:** ^1^ Centre for Biomechanics and Rehabilitation Technologies, Science Centre Staffordshire University Stoke on Trent UK; ^2^ Royal Wolverhampton NHS Trust Wolverhampton UK; ^3^ Scolio Centar Novi Sad Serbia; ^4^ University Hospitals of North Midlands NHS Trust Stoke on Trent UK

**Keywords:** adolescents, patient reported outcome measures, questionnaire design, scoliosis, validation

## Abstract

**Introduction:**

Adolescent Idiopathic Scoliosis (AIS) is a structural spinal deformity with implications for health‐related quality of life (HR‐QoL). The Scoliosis Research Society‐22 revised (SRS‐22r) questionnaire is the standard for HR‐QoL assessment. However, studies have identified limitations with the SRS‐22r, including content and face validity issues, reliability concerns, and language appropriateness. This study aimed to develop and validate a patient‐reported questionnaire, the Staffordshire Questionnaire for Adolescent Idiopathic Scoliosis (SQ‐AIS), to assess the impact of AIS on HR‐QoL.

**Methods:**

The SQ‐AIS comprises six domains: general health, pain, function/activity, self‐image/appearance, mental health, and intervention. Individuals with AIS aged 10–19 years and clinicians from a range of countries with expertise in AIS contributed to the testing process. Face validity and clinical applicability were assessed using Likert scales, while content validity was evaluated through a categorical binary variable (yes/no).

**Results:**

Involving 8 AIS patients and 43 clinicians, face validity scores demonstrated an acceptable level of understanding (≥ 4/5) for both individuals with AIS and clinicians. Most individuals with AIS (85.71%) and clinicians (80.95%) affirmed that the questionnaire sufficiently covers various aspects of scoliosis, indicating a satisfactory level of content validity. Ratings for applicability to clinical practice indicated an acceptable level of practical relevance (≥ 4/5).

**Discussion and Conclusion:**

The SQ‐AIS emerges as a valid and promising tool to overcome existing challenges in AIS‐related outcome assessment. Pending further validation studies, the favorable reception from the international community of clinicians suggests its potential as a new benchmark for evaluating AIS impact on HR‐QoL and monitoring scoliosis management.

## 1 Introduction

Adolescent idiopathic scoliosis (AIS) is the most common form of structural spinal deformities [1] and is characterised by a three‐dimensional structural lateral deviation of the spine (Cobb angle) exceeding 10° [2]. It affects between 1% and 3% of children aged between 10 and 16 years [1], with higher prevalence among females and the female‐to‐male ratio increasing with the severity of the spinal curve [3]. While AIS often shows no symptoms, it can result in physical deformities including chest wall abnormalities, rib prominence, abnormality in shoulder height and waist line, and truncal shift [4]. Incorrect postures may impact self‐image and contribute to psychological disturbances like lack of self‐esteem and depression [5]. Scoliosis can also lead to back pain, limitations in functional activities (e.g., standing and walking) and, more rarely, pulmonary impairments (e.g., shortness of breath) [1]. These symptoms can significantly impact the health‐related quality of life (HR‐QoL) of individuals living with this condition [6].

The impact of AIS on individuals and the measurement of treatment outcomes on HR‐QoL is commonly assessed using patient‐reported outcome measures (PROMs). The most frequently used PROM to assess HR‐QoL in individuals with scoliosis is the Scoliosis Research Society (SRS) questionnaire in its multiple variants (22, 22 revised, 23, 24, and 30) [7]. Currently, the SRS has encouraged clinicians to use the SRS‐22 revised version (SRS‐22r) of the questionnaire, making it the most widely used SRS version [7]. The SRS‐22r has undergone translation and adaptation into multiple languages [8, 9, 10], with its psychometric properties widely investigated [8, 9, 10, 11].

Although the SRS‐22r ample use may suggest acceptance within clinical and research practice, previous studies have highlighted multiple limitations of the questionnaire. Notably, participants involved in studies assessing the psychometric properties of the SRS‐22r were adults, raising concerns about the generalisability of these findings to the AIS population [12]. The SRS‐22r showed significant ceiling effects (20%–44%) [13], compromising its content validity and reliability. Content validity concerns are amplified by the use of previous questionnaires designed for assessing surgical treatments in adults with scoliosis during the SRS‐22 development process [14]. Additionally, the inclusion of questions from the SF‐36 survey in the mental health domain poses a further issue, as these questions are nonspecific to AIS and are tailored for an adult population rather than a pediatric one. Recent qualitative research has highlighted that the terminology used in the SRS‐22r does not align with the language commonly employed by individuals with AIS, indicating poor face validity [15]. Furthermore, the assessment questions communicate a distinctively negative perspective on scoliosis, potentially contributing to a stigmatised perception of the condition. Additionally, these questions exhibit a subtle yet noticeable leading bias, which may influence respondents' perceptions and subsequent responses. Furthermore, qualitative responses from individuals with AIS have suggested a lack of content validity, as the SRS‐22r fails to adequately capture the lived experiences of adolescents with AIS and the impact of scoliosis on their HR‐QoL [15]. Notably, symptoms such as stiffness, hip and shoulder pain, body asymmetry, the impact of scoliosis on activities of daily living, and the psychological impact of AIS are not assessed by the SRS‐22r [15]. Consequently, there is a clear need for a new PROM tailored to AIS that comprehensively captures the broad impact of the condition and employs language that resonates with the affected population.

This study aimed to develop and validate a PROM, named the Staffordshire Questionnaire for Adolescent Idiopathic Scoliosis (SQ‐AIS), to assess the impact of AIS on the HR‐QoL of people living with the condition.

## 2 Methods

### 2.1 Stage 1: Questionnaire Development

**Figure 1:**
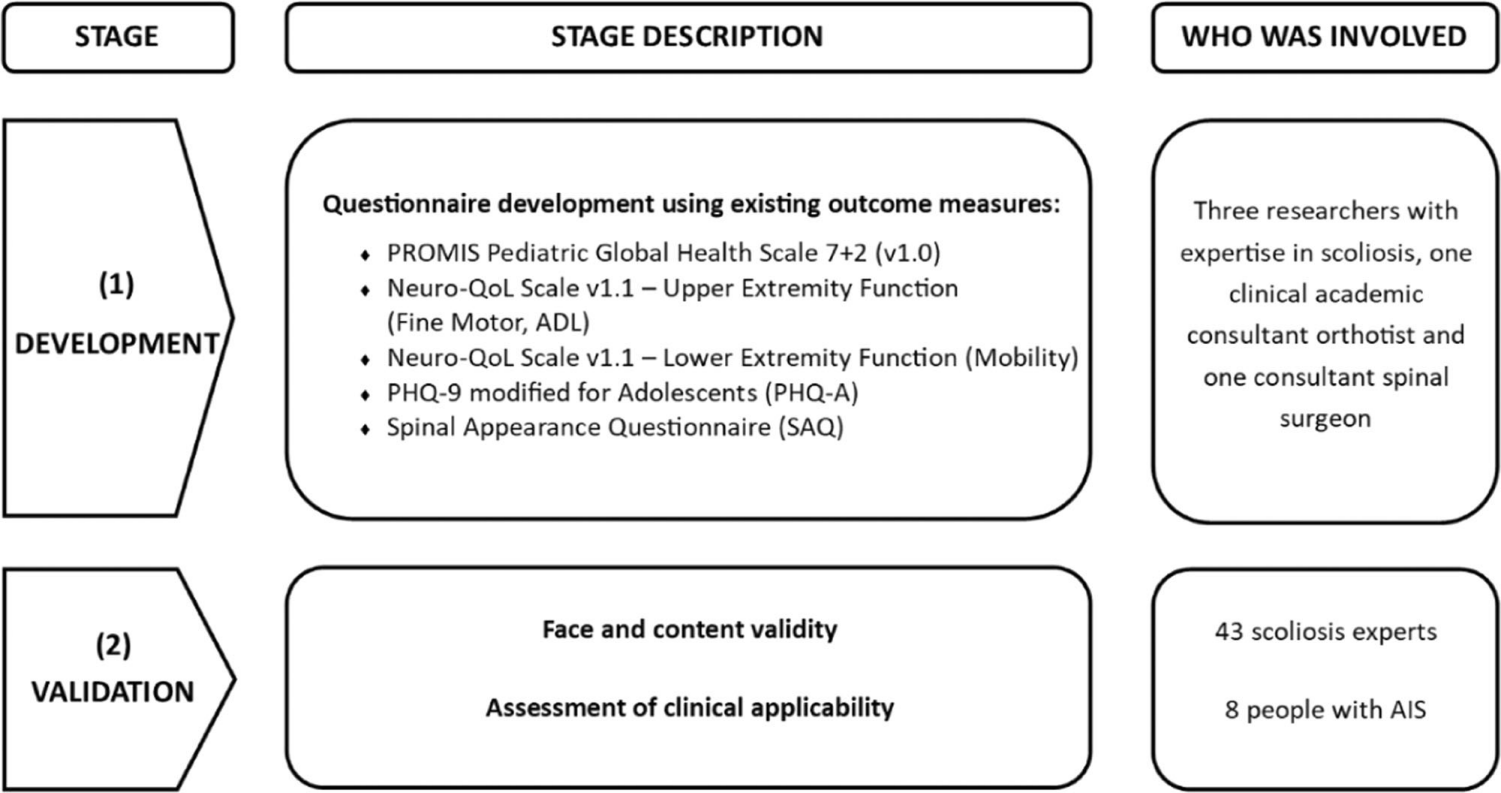
Development and validation of the SQ‐AIS. ADL, activities of daily living; AIS, adolescent idiopathic scoliosis; Neuro‐QoL, quality of life in neurological disorders; PHQ‐9, Patient Health Questionnaire‐9.

The SQ‐AIS was designed by three UK‐based researchers with expertise in scoliosis, one clinical academic consultant orthotist, and one consultant spinal surgeon. It incorporates elements from outcome measures investigating aspects of HR‐QoL and global health in pediatric and adolescent populations (Figure 1). The initial version of the questionnaire, subject to testing and validation, comprised six domains: general health (13 items), pain (17 items), function/activity (22 items), self‐image/appearance (10 items), mental health (8 items), and intervention (31 items). The score for each of the five domains (scores are not attributed to the intervention section) is calculated as the mean of all the answered questions within that specific domain. The overall score is then calculated as the mean of all answered questions across the five domains, with higher scores indicating better HR‐QoL.

### 2.2 Stage 2: Testing

This process involved two groups: (1) individuals with a confirmed diagnosis of AIS participating in the International Schroth Scoliosis Therapy (ISST) Camp, a comprehensive multiday treatment program for scoliosis held at the Scolio Centar in Novi Sad (Serbia), and (2) a convenience sample comprising clinicians and researchers with expertise in scoliosis.

The first group was recruited from various ISST Camps held during summer 2021. At the camps, individuals with AIS were informed that the research team was seeking feedback on a newly developed PROM. Eligible participants included those who met the following criteria: (1) age between 10 and 19 years, aligning with the World Health Organization's definition of adolescence [16], (2) ability to provide informed consent for both participants and their parent(s) or legal guardian(s), and (3) proficiency in English for either the patient or their parent(s) or legal guardian(s).

This second group was drawn from a multinational group of attendees of the 2021 International Scoliosis Symposium (ScoSym) (18–19 September 2021, Novi Sad, Serbia). During the conference, attendees were informed that the research team was seeking feedback on a newly developed PROM. Interested participants were encouraged to approach the research team. Any conference attendee who expressed willingness to participate was considered eligible for inclusion in this study, given that their conference attendance indicated a reasonable level of competence in the field of scoliosis.

Participants were provided a hard copy of the SQ‐AIS and rated its face and content validity, and applicability to clinical practice and overall usability. The clinicians participating in the testing process were given the option to disclose their demographic information or remain anonymous.

### 2.3 Assessment of Face Validity

Face validity was assessed using a 5‐point Likert scale ranging from 1 (No, very difficult) to 5 (Yes, very easy). Three areas were assessed under the face validity construct: (a) the extent to which the questionnaire was easy to understand for respondents, (b) the degree of clarity of anticipated comprehension for individuals with AIS aged 10–17, and (c) the degree of anticipated clarity for those aged 18 years or older. Both individuals with AIS and clinicians with expertise in scoliosis participated in the face validity assessment.

### 2.4 Assessment of Content Validity

Content validity was assessed through a categorical global content validity judgment, wherein respondents indicated “yes” *(relevant)*, “unsure,” or “no” *(not relevant)*. Given the exploratory nature of this study, the evaluation focused on content validity at a scale level, aiming to understand the overall relevance of the outcome measure to scoliosis assessment rather than analysing individual items.

Both individuals with AIS and clinicians with expertise in scoliosis participated in the content validity assessment. The evaluation of content validity involved calculating the overall percentage of individuals with AIS and clinicians who considered the outcome measure sufficiently comprehensive in addressing various clinical aspects of scoliosis. Respondents were encouraged to provide qualitative feedback through open‐text questions, if any gaps in clinical assessment components were identified.

### 2.5 Assessment of Clinical Applicability

The applicability to the clinical practice section was completed by clinicians only. This section consisted of two areas: (a) perceived usefulness in clinical practice and (b) the likelihood of using the questionnaire in clinical practice. Perceived usefulness in clinical practice was assessed using a 4‐point Likert scale, ranging from 4 (*Yes, very helpful*) to 1 (*No, not helpful at all*). The likelihood to use this questionnaire in clinical practice was evaluated with a 5‐point Likert scale, ranging from 5 (*Yes, definitely*) to 1 (*No, definitely not*). Respondents uncertain about using the questionnaire in clinical practice were asked to share their reasons. Additionally, an open‐text question was included to gather any further qualitative feedback to refine the questionnaire.

### 2.6 Data Analysis

Statistical analysis was conducted using Statistical Package for Social Science (SPSS) software (IBM SPSS Statistics Version 25). The data were tested for normality using graphical methods such as histograms and Shapiro–Wilk test, where *p* > 0.05 were considered significant [17]. For face validity and applicability to clinical practice, continuous data were summarised as mean and standard deviation (SD) if normally distributed and as median and interquartile ranges (IQR) when non‐normally distributed. All categorical variables and content validity were presented as percentages.

## 3 Results

### 3.1 Participant Demographics

Forty‐three clinicians and eight individuals with AIS took part in the testing and validation process. Twenty‐nine clinicians provided their demographic information, while 14 participated anonymously. Clinician demographics revealed a wide geographical representation, including central and eastern European countries (Serbia [*n* = 10], Croatia [*n* = 7], Greece and Ukraine [*n* = 3 each], Poland [*n* = 2], Albania, Bosnia and Herzegovina, Germany, and Turkey [*n* = 1 each]). Most clinicians were physiotherapists (*n* = 23), other healthcare professions represented were prosthetic and orthotic technicians (*n* = 2), medical doctors specialised in physiatry (*n* = 1), and pediatric and orthopedic surgery (*n* = 1). Six clinicians also disclosed their certification as Schroth‐certified therapists. Professional experience of scoliosis ranged from 1 to 40 years among participants, with a median of 4 years (IQR = 6).

Participants with AIS were mostly females (7F:1M) and their median age was 15.5 years (IQR = 2.5). Participants presented diverse spinal curvature patterns, including an equal number of major thoracic and lumbar curves, alongside variations in secondary curves observed in the lumbar, thoracic, and cervicothoracic regions. Thoracic curve angles ranged from 30° to 50°, while lumbar curves ranged from 21° to 52°.

While this sample size may seem modest, it aligns with established standards in the field. The international Delphi study by Terwee et al. [18] identified seven participants as the minimum threshold for achieving a very good rating in content validity testing for patient‐reported outcomes. Furthermore, comparable studies in scoliosis research, such as Alamrani et al. [19], typically aim to involve between 10 and 15 participants with AIS, with recruitment numbers similar to those in this study. Thus, the inclusion of eight individuals in this study appears to be adequate for its intended purpose.

### 3.2 Face Validity

The face validity results (Table 1) revealed median scores of ≥ 4 across the domains for both individuals with AIS and clinicians. These scores fall within the “yes, very easy” to “yes, easy” categories, indicating an acceptable level of face validity.

**Table 1:** Face validity, content validity, and applicability to clinical practice of the SQ‐AIS.

Domain	People with AIS (*n* = 8)	Clinicians (*n* = 43)
**Face validity**		
Easily understandable	5 (0.5)	5 (0.5)
Easily understandable for individuals aged 10–17	4 ± 0.71	4 (1)
Anticipated ease for individuals 18 and older to understand	5 (0.5)	5 (0.5)
**Content validity**		
Provides adequate breadth of scoliosis clinical aspects^a^	Yes (85.71%, *n* = 6) No (14.29%, *n* = 1)	Yes (80.95%, *n* = 34) Unsure (14.29%, *n* = 6) No (4.76%, *n* = 2)
**Applicability to clinical practice**		
Perceived usefulness in clinical practice^b^	—	4 (0.5)
Likelihood to use the questionnaire in clinical practice^b^	—	4.5 (0.5)

*Note:* Face validity and applicability to clinical practice were calculated as the median Likert scale ranking value. Face validity 5‐point Likert scale ranking ranged from 1 (*No, very difficult*) to 5 (*Yes, very easy*), while applicability to clinical practice 4‐point Likert scale ranking ranged from 4 (*Yes, very helpful*) to 1 (*No, not helpful at all*). The values for all domains except “Easily understandable for individuals aged 10–17” are expressed as median (IQR, interquartile range).

a Based on 7 individuals with AIS and 42 clinicians.

b Based on 42 clinicians.

### 3.3 Content Validity

All bar one of the individuals with AIS (85.71%, *n* = 6) and most clinicians (80.95%, *n* = 34) considered the questionnaire to provide adequate breadth of scoliosis clinical aspects (see Table 1). These findings suggest a satisfactory level of content validity for the questionnaire.

### 3.4 Applicability to Clinical Practice

The calculated median, derived from the statement parameters, consistently yielded positive scores (≥ 4), signifying an acceptable level of applicability within the two identified domains (Table 1).

### 3.5 Qualitative Feedback

Participants found the questionnaire accessible and commended its detailed, careful, and accurate structure. The perceived strengths include its concreteness, comprehensiveness, and well‐organised format, addressing crucial aspects often discussed by patients, sometimes inadvertently. Nevertheless, valuable insights were gathered through the qualitative component of the survey. The most common suggested improvements included incorporating figures to illustrate the perceived appearance of the spine and expanding on interventions for scoliosis management, including potential surgical procedures. In response to the feedback received, the questionnaire was revised, and the updated version of the questionnaire, along with the scoring system, is available in File S1.

## 4 Discussion

Prior attempts to design alternative PROMs to address deficiencies of the SRS‐22r have proven unsuccessful [20, 21], resulting in an unmet need for a valid and reliable PROM capable of replacing the SRS‐22r. Consequently, the SQ‐AIS was developed with the explicit aim of rectifying these concerns and filling existing gaps for AIS HR‐QoL assessment. The overarching goal was to provide a comprehensive tool facilitating the identification of diverse HR‐QoL domains possibly affected in individuals with AIS, ultimately guiding the selection of interventions and facilitating the monitoring of the overall management of these individuals.

The face validity results were notably satisfactory, as both the individuals with AIS and clinicians confirmed the SQ‐AIS's ease of understanding for themselves, as well as for potential adolescent and adult individuals with idiopathic scoliosis. Qualitative feedback further strengthened this observation, with participants consistently acknowledging the questionnaire's simplicity and well‐organized structure. While all assessed domains achieved satisfactory levels of face validity, it is worth noting that the item performing least effectively for both the individuals with AIS and clinicians was the perceived ease of understanding the questionnaire for individuals with AIS aged 10–17. This may suggest that younger adolescents with idiopathic scoliosis might find the questionnaire slightly more challenging to comprehend compared to older individuals in the adolescence age range and adults. However, this does not pose a significant concern for broader applicability in the AIS population, as the questionnaire has been generally considered comprehensible for them by both people of the same age range and clinicians. Additionally, the presence of parents or guardians during pediatric consultations can facilitate understanding, mitigating potential comprehension challenges for younger participants. Furthermore, these findings indicate that the SQ‐AIS, in its current form, is easily understood by adults with the condition. Therefore, with appropriate adaptations tailored to the adult population, a revised format of the SQ‐AIS could be effectively applied to adults living with idiopathic scoliosis.

Recognising the limitations of the SRS‐22r in fully capturing the impact of AIS on HR‐QoL, the SQ‐AIS was designed to serve as an alternative tool that offers a more comprehensive and multidimensional patient PROM, which can also be used as a one‐time assessment. Furthermore, in contrast to the SRS‐22r, which was originally designed for the adult population with scoliosis [14], our approach integrated measures with robust psychometric properties tailored specifically for use in pediatric populations. This thorough and targeted methodology likely contributed to the positive results in terms of content validity. This validation was further confirmed by qualitative feedback from study participants who not only recognised the questionnaire's comprehensiveness, but also acknowledged its inclusion of crucial aspects related to AIS, highlighting the clinical relevance of the questionnaire. Notably, while sharing four domains with the SRS‐22r (function, pain, self‐image, and mental health), our questionnaire includes a novel intervention section. Following qualitative feedback, this section underwent expansion to encompass surgical management, enhancing its comprehensiveness. This innovative feature proves invaluable to healthcare professionals, offering a better understanding of the diverse spectrum of AIS symptomatology. It facilitates effective monitoring of AIS management and aids in comprehending how conservative or surgical interventions may impact HR‐QoL of individuals with AIS. The SQ‐AIS has the potential to contribute to the enhanced effectiveness of AIS management, improved health outcomes and HR‐QoL for individuals with AIS.

We would also like to highlight that we have adopted a robust approach to handling missing data in the SQ‐AIS. The threshold of 20% for missing data per section is well within accepted standards and compares favorably to other established tools such as the SRS‐22r, which allows up to 40% missing data in each section. The SQ‐AIS's design, with more items per domain than the SRS‐22r, further mitigates the potential impact of missing data on overall scoring. This aligns with research by Sloan et al. [22], suggesting that missing data up to 20% is unlikely to significantly affect study findings. Consequently, the SQ‐AIS demonstrates an advantage in data completeness and reliability compared to existing tools, enhancing the validity of the results obtained.

Clinicians from a range of countries positively received the SQ‐AIS, recognising its potential as a valuable tool for exploring the extensive impact of AIS on HR‐QoL. Furthermore, these clinicians expressed a high likelihood of incorporating SQ‐AIS into their own clinical practices. The international recognition it received suggests that SQ‐AIS has the potential to be widely adopted as a PROM for assessing the impact of AIS on HR‐QoL. However, it is essential to note that its clinical application awaits further validation studies, which are currently underway. These ongoing studies aim to explore additional psychometric properties (e.g., construct and convergent validity, test–retest reliability, and internal consistency) and assess how it compares to existing gold standards for evaluating the impact of AIS on HR‐QoL.

The proposed questionnaire, though lengthy, serves as a unified assessment tool rather than multiple instruments used across various centers. Its widespread adoption will aid in understanding global incidence, in contrast to the current practice of using localised questionnaires and assessment tools which differ from country to country, making comparisons challenging.

This study has several strengths. The initial version of the SQ‐AIS was developed by researchers and clinicians with scoliosis expertise. Subsequently, a substantial number of clinicians with expertise in scoliosis from a range of countries participated in the testing process, offering invaluable qualitative feedback that played a pivotal role in refining the questionnaire further. Incorporating the perspectives of clinicians from a range of countries with diverse backgrounds ensured a thorough and robust testing and validation process. Furthermore, the SQ‐AIS received validation not only from clinicians but also from individuals with lived experience of the condition, which highlights its effectiveness in capturing the multidimensional aspects of AIS.

In acknowledging study limitations, it is essential to note that all participants with AIS were from Serbia, raising the consideration that individuals with AIS from varying cultural, ethnic, and socioeconomic backgrounds might perceive the questionnaire differently. Additionally, the scoliosis questionnaire was available only in English. Although participants with AIS were proficient in the English language, some assistance from parents and healthcare professionals was provided during questionnaire completion, potentially influencing responses. Individuals with AIS were not directly involved in the questionnaire's development; however, their feedback was sought during the testing phase and subsequently incorporated. Another limitation of this study is that we did not measure the average time taken to complete the questionnaire. The length of the questionnaire could potentially be refined. However, it is important to note that the current length is a result of the tool's comprehensive nature, designed to capture a wide range of relevant data points.

## 5 Conclusion

The SQ‐AIS demonstrated satisfactory levels of face and content validity according to individuals with AIS and clinicians from a range of countries. The comprehensiveness, well‐structured design, and ease of understanding of the newly developed PROM was acknowledged. The SQ‐AIS's inclusion of an intervention section enhances its versatility, enabling effective monitoring of AIS symptoms, both conservative and surgical interventions, and their subsequent impact on HR‐QoL. The positive reception from clinicians from a range of countries highlights the SQ‐AIS's potential as a valuable instrument for evaluating AIS impact on HR‐QoL internationally. As the SQ‐AIS undergoes further validation, it emerges as a promising candidate for a standalone measure, providing a comprehensive assessment of AIS impact on HR‐QoL and serving as a valuable tool for the ongoing monitoring of scoliosis management.

## Author Contributions

**Enza Leone:** writing–review and editing, validation, writing–original draft, data curation, formal analysis. **Nachiappan Chockalingam:** conceptualization, investigation, methodology, supervision, writing–review and editing, project administration, funding acquisition, data curation, formal analysis. **Robert Needham:** methodology, writing–review and editing. **Aoife Healy:** methodology, writing–review and editing, data curation, formal analysis. **Nicola Eddison:** methodology, writing–review and editing. **Nikola Jevtic:** investigation, project administration, writing–review and editing, resources. **Vinay Jasani:** methodology, writing–review and editing, resources.

## Ethics Statement

Ethical approval was obtained from the Faculty of Sport and Physical Education at the University of Novi Sad (Reference number: 49‐02‐04/2023) and the East Midlands—Derby Research Ethics Committee (REC reference number: 11/WM/0037). Written informed consent was obtained from study participants.

## Conflicts of Interest

E.L., R.N., A.H., and V.J. have no other affiliations or interests apart from their core affiliation within their academic or clinical practice. N.E. is the chair of the British Association of Prosthetists and Orthotists. N.C. is the current president of the International Society of Spinal Deformities and N.J. is a physiotherapist and also offers Schroth Exercise training programs. None of the authors declare any direct conflict of interest with the contents of this manuscript.

## Transparency Statement

The lead author, Chockalingam (manuscript guarantor), affirms that this manuscript is an honest, accurate, and transparent account of the study being reported; that no important aspects of the study have been omitted; and that any discrepancies from the study as planned (and, if relevant, registered) have been explained.

## Supporting information

Supporting information.

## Data Availability

The data that support the findings of this study are available from the corresponding author upon reasonable request.
