# Supplementary material for: Development and Preliminary Testing of the Staffordshire Questionnaire for Adolescent Idiopathic Scoliosis (SQ‐AIS): Content and Face Validity
Source: Health Sci Rep. 2024 Nov 22;7(11):e70213. doi: 10.1002/hsr2.70213 (PMC11582472; doi:10.1002/hsr2.70213)
Supplement: Supplementary file 1 — Supporting information. [file HSR2-7-e70213-s001.docx]

**Supplementary file 1. Staffordshire Questionnaire for Adolescent Idiopathic Scoliosis (SQ-AIS) and its scoring system**

**Staffordshire Questionnaire for Adolescent Idiopathic Scoliosis (SQ-AIS)**

**Please complete this questionnaire if you are between 10-19 years old and have been told that you have idiopathic scoliosis.**

**Section 1. General health**

**Q1. Other than scoliosis, have you been diagnosed with any other medical condition(s)? Please put an ‘X’ in the box which applies to you:**

- Yes
- No

**Q1.1.** If yes, what medical condition(s) do you have?

| **Q2 Please put an ‘X’ in the box most relevant to you:** | Excellent | Very good | Good | Fair | Poor |
| --- | --- | --- | --- | --- | --- |
| **Q2.1.** In general, would you say your health is? |  |  |  |  |  |
| **Q2.2.** In general, would you say your day-to-day happiness is? |  |  |  |  |  |
| **Q2.3.** In general, how would you rate your physical health? |  |  |  |  |  |
| **Q2.4.** In general, how would you rate your mental health, including your mood and your ability to think? |  |  |  |  |  |

|  | Never | Rarely | Sometimes | Often | Always |
| --- | --- | --- | --- | --- | --- |
| **Q3.1.** How often do you have fun with your friends? |  |  |  |  |  |
| **Q3.2.** How often you feel frustrated |  |  |  |  |  |
| **Q3.3.** How often do you feel sad? |  |  |  |  |  |

|  | Once per  week | One per  fortnight | Once per  month | Rarely | Never | Not  Applicable |
| --- | --- | --- | --- | --- | --- | --- |
| **Q4.** How often do you miss Physical  Education (PE)? |  |  |  |  |  |  |

| **Q5. In the past 7 days…** | Never | Almost  never | Sometimes | Often | Almost  Always |
| --- | --- | --- | --- | --- | --- |
| **Q5.1.** I got tired easily |  |  |  |  |  |
| **Q5.2.** I had trouble sleeping due to pain |  |  |  |  |  |
| **Q5.3.** I have felt frustrated or had other negative emotions other than pain |  |  |  |  |  |

**Section 2. Pain**

**Q6.** As a result of your scoliosis how often do you experience pain in the following areas of the body at **rest in the last 6 months**:

|  | None of the  time | A little of the  time | Some of the  time | Most of the  time | All of the  time |
| --- | --- | --- | --- | --- | --- |
| **Q6.1.** Head and/or neck |  |  |  |  |  |
| **Q6.2.** Chest/Back/Stomach |  |  |  |  |  |
| **Q6.3**. Arms and/or hand |  |  |  |  |  |
| **Q6.4**. Legs and/or feet |  |  |  |  |  |

**Q7.** As a result of your scoliosis how often do you experience pain in the following areas of the body during **physical activity in the last 6 months**:

|  | None of the  time | A little of the  time | Some of the  time | Most of the  time | All of the  time |
| --- | --- | --- | --- | --- | --- |
| **Q7.1**. Head and/or neck |  |  |  |  |  |
| **Q7.2.** Chest/Back/Stomach |  |  |  |  |  |
| **Q7.3.** Arms and/or hand |  |  |  |  |  |
| **Q7.4.** Legs and/or feet |  |  |  |  |  |

**Q8**. As a result of your scoliosis how often do you experience pain in the following areas of the body during **rest in the last month**:

|  | None of the  time | A little of the  time | Some of the  time | Most of the  time | All of the  time |
| --- | --- | --- | --- | --- | --- |
| **Q8.1.** Head and/or neck |  |  |  |  |  |
| **Q8.2.** Chest/Back/Stomach |  |  |  |  |  |
| **Q8.3**. Arms and/or hand |  |  |  |  |  |
| **Q8.4**. Legs and/or feet |  |  |  |  |  |

**Q9.** As a result of your scoliosis how often do you experience pain in the following areas of the body during **physical activity in the last month**:

|  | None of the  time | A little of the  time | Some of the  time | Most of the  time | All of the  time |
| --- | --- | --- | --- | --- | --- |
| **Q9.1.** Head and/or neck |  |  |  |  |  |
| **Q9.2.** Chest/Back/Stomach |  |  |  |  |  |
| **Q9.3.** Arms and/or hand |  |  |  |  |  |
| **Q9.4**. Legs and/or feet |  |  |  |  |  |

**Q10.** As a result of your scoliosis how many days have you taken off from work/school in the last 12 months?

- None ☐ Number of days …………….

**Section 3. Function / activity**

| **Q11**. In the past 7 days………….. | With no  trouble | With a little  trouble | With some  trouble | With a lot of trouble | Not able to  do |
| --- | --- | --- | --- | --- | --- |
| **Q11.1**. I was able to take off my socks |  |  |  |  |  |
| **Q11.2.** I was able to put on and fasten my pants/trousers by myself |  |  |  |  |  |
| **Q11.3.** I was able to button and unbutton my shirt |  |  |  |  |  |
| **Q11.4**. I was able to wipe myself thoroughly after using the toilet |  |  |  |  |  |
| **Q11.5**. I was able to pull my pants back up after using the toilet. |  |  |  |  |  |
| **Q11.6.** I was able to take a shower/bath by myself |  |  |  |  |  |
| **Q11.7.** I was able to get out of bed by myself |  |  |  |  |  |
| **Q11.8.** I can reach the top shelf in a cupboard of my height comfortably |  |  |  |  |  |
| **Q11.9.** I am able to join in activities with my friends |  |  |  |  |  |

| In the past 7 days………… | With no  trouble | With a little  trouble | With some  trouble | With a lot of trouble | Not able to  do |
| --- | --- | --- | --- | --- | --- |
| **Q11.10.** I could get down on my knees without holding on to something |  |  |  |  |  |
| **Q11.11.** I could keep up when I played with my friends |  |  |  |  |  |
| **Q11.12.** I could walk for 15 minutes |  |  |  |  |  |
| **Q11.13.** I could walk between rooms |  |  |  |  |  |
| **Q11.14**. I could get on and off the toilet without using my arms |  |  |  |  |  |
| **Q11.15.** I could get on and off a low chair |  |  |  |  |  |
| **Q11.16.** I could get up from the floor by myself |  |  |  |  |  |
| **Q11.17.** I could sit on a bench without support for 15 minutes |  |  |  |  |  |
| **Q11.18.** I could stand on my tiptoes to reach for something |  |  |  |  |  |
| **Q11.19.** I could stand on my tiptoes to put something (e.g., a bag of sugar) on a shelf |  |  |  |  |  |
| **Q11.20.** I could walk on slightly uneven surfaces (such as cracked pavement/footpath) |  |  |  |  |  |
| **Q11.21.** I could walk on rough, uneven surfaces (such as lawns, gravel driveway) |  |  |  |  |  |
| **Q11.22.** I could walk up and down ramps or hills |  |  |  |  |  |

**Section 4. Self-image / appearance**

**Q12.** Please look at the following images and tick the circle underneath the image which looks most like you:


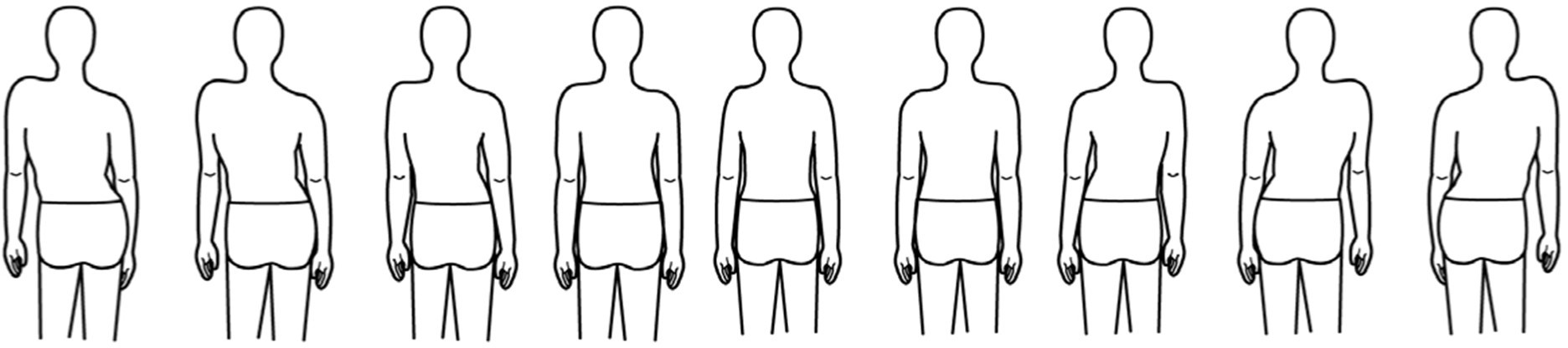

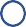

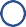

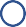

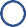

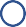

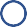

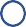

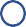

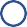


**Q13.** How would you rate the way you look?

Very bad Bad Fair Good Very good


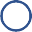

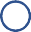

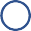

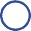

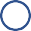


**Q14.** Do you think the following statements are true?

Put an “X” in the box beneath the answer that best describes what you think.

|  | Not True | A little true | Somewhat true | Fairly true | Very true |
| --- | --- | --- | --- | --- | --- |
| **Q14.1.** I think I look different to my friends |  |  |  |  |  |
| **Q14.2.** I want to change the way I look |  |  |  |  |  |
| **Q14.3.** I want to look better in clothes |  |  |  |  |  |
| **Q14.4.** The way I look makes me feel sad, unhappy or angry |  |  |  |  |  |
| **Q14.5.** The way I look stops me from joining in with my friends |  |  |  |  |  |
| **Q14.6.** I am always thinking about the way I look |  |  |  |  |  |
| **Q14.7.** When I meet new people, I wonder what they think about the way I look |  |  |  |  |  |
| **Q14.8.** The way someone looks is not important |  |  |  |  |  |

**Section 5. Mental health**

**Q15.** How often have you experienced the following symptoms during the past two months? For each symptom put an “X” in the box beneath the answer that best describes how you have been feeling.

|  | Not at all | Several days | More than half  the days | Nearly every day |
| --- | --- | --- | --- | --- |
| **Q15.1.** Feeling down, unhappy,  irritable, or hopeless? |  |  |  |  |
| **Q15.2.** Little interest or pleasure in  doing things? |  |  |  |  |
| **Q15.3.** Trouble falling asleep, staying asleep, or sleeping too much? |  |  |  |  |
| **Q15.4.** Poor appetite, weight loss, or overeating? |  |  |  |  |
| **Q15.5.** Feeling tired, or having little  energy? |  |  |  |  |
| **Q15.6.** Feeling bad about yourself? |  |  |  |  |
| **Q15.7.** Trouble concentrating on things like school, work, reading, or watching TV? |  |  |  |  |
| **Q15.8.** Moving or speaking so slowly that other people could have noticed? Or the opposite – being so fidgety or restless that you were moving around a lot more than usual? |  |  |  |  |

**Section 6. Intervention**

**Q16.** Do you take any medication due to your scoliosis?

- Yes ☐ No

**Q16.1**. If yes, which one of the following best describes your medication usage due to your scoliosis? *This question can be completed by a parent/guardian if required.*

|  | Daily | Every 2-4 days | Every 5-7 days | Monthly | Occasionally |
| --- | --- | --- | --- | --- | --- |
| Medication prescribed by  my doctor |  |  |  |  |  |
| Medication I get over the  counter |  |  |  |  |  |
| Herbal remedies |  |  |  |  |  |
| Other (please specify) |  |  |  |  |  |
| Other (please specify) |  |  |  |  |  |
| Other (please specify) |  |  |  |  |  |

**Q17.** Are you currently wearing a brace for your scoliosis?

- - Yes
  - No

**Q17.1.** If yes, how long do you usually wear your brace for? Put a “X” under the one applies to you:

| I choose not to wear it | Less than 4 hours per day | 4-8 hours per day | 9-12 hours per day | 13-16  hours per day | 17-24  hours per day |
| --- | --- | --- | --- | --- | --- |
|  |  |  |  |  |  |

| **Q18.** If yes, do you think the following statements are true? Put an “X” in the box beneath the answer that best describes what you think about your brace: | Strongly agree | Agree | Undecided | Disagree | Strongly disagree |
| --- | --- | --- | --- | --- | --- |
| **Q18.1.** I feel uncomfortable by the appearance of my body in the brace. |  |  |  |  |  |
| **Q18.2.** I feel unattractive in my brace |  |  |  |  |  |
| **Q18.3.** It is hard for me to be myself with my brace. |  |  |  |  |  |
| **Q18.4.** I feel uncomfortable in situations where other people can see my brace. |  |  |  |  |  |
| **Q18.5.** I don't feel embarrassed when people see my brace. |  |  |  |  |  |
| **Q18.6.** I avoid body contact so that no-one knows that I wear a brace. |  |  |  |  |  |
| **Q18.7.** When deciding what kind of clothes to wear or how to wear my hair, I try to make sure my brace is hidden. |  |  |  |  |  |
| **Q18.8.** I don't feel embarrassed to show my brace to people close to me (parents, friends and school- friends). |  |  |  |  |  |
| **Q18.9.** Because of my brace I avoid activities/hobbies, which otherwise I love to  do. |  |  |  |  |  |

**Q19.** Are you currently receiving physiotherapy to treat your scoliosis?

- Yes
- No

**Q19.1.** If yes, do you think the following statements are true? Put an “X” in the box beneath the answer that best describes what you think about the physiotherapy you are currently doing.

|  | Strongly  agree | Agree | Undecided | Disagree | Strongly  disagree |
| --- | --- | --- | --- | --- | --- |
| **Q19.1.1.** Physiotherapy is important in  the management of my scoliosis. |  |  |  |  |  |
| **Q19.1.2.** Physiotherapy decreases my pain.  If you selected “strongly agree” or “agree”, state how/which exercises below: |  |  |  |  |  |
| **Q19.1.3.** Physiotherapy improves my sleep. |  |  |  |  |  |
| **Q19.1.4.** Physiotherapy increases my feelings of well-being. |  |  |  |  |  |
| **Q19.1.5.** Physiotherapy improves my fitness  If you selected “strongly agree” or “agree”, state how/which exercises below: |  |  |  |  |  |

**Q20.** Have you had surgery to treat your scoliosis?

- Yes
- No

**Q20.1.** If yes did you have:

Fusion surgery

Non fusion surgery (eg VBT)

**Q20.2.** If yes, how long ago did you have surgery?

< 6 months 6-12 months > 12 months

**Q21.** If yes, do you think the following statements are true? Put an “X” in the box beneath the answer that best describes what you think about the surgery that you have had.

|  | Strongly  agree | Agree | Undecided | Disagree | Strongly  disagree |
| --- | --- | --- | --- | --- | --- |
| **Q21.1.** Surgery was an important part of  the management of my scoliosis. |  |  |  |  |  |
| **Q21.2.** Surgery has helped to decrease my pain. |  |  |  |  |  |
| **Q21.3.** I am able to sleep better than before. |  |  |  |  |  |
| **Q21.4.** Surgery has increased my feelings of well-being. |  |  |  |  |  |
| **Q21.5**. This treatment has improved my fitness and physical activity. |  |  |  |  |  |
| **Q21.6.** Surgery has improved my appearance and body image |  |  |  |  |  |
| **Q21.7.** Surgery has stopped me doing activities that I want to do long term |  |  |  |  |  |
| **Q21.8**. I feel surgery has made a positive impact on my scoliosis |  |  |  |  |  |
| **Q21.9**. I am glad that I had surgery for my scoliosis |  |  |  |  |  |

**Staffordshire Questionnaire for Adolescent Idiopathic Scoliosis (SQ-AIS) – scoring instructions**

- Score 5 for best, 1 for worst.
- Mental health questions (from Q15.1 to Q15.8) are scored as follows: ‘Not at all’=5, ‘Several days’=3.75, ‘More than half the days’=2.5, ‘Nearly every day’=1.25
- Following questions don’t need to be scored: Q.1., Q1.1. Q10, entire section 6 (intervention) (Q16 to Q21.9)
- Q3.2., Q3.3., Q4, Q5.1., Q5.2. and Q5.3: Never=5, always or almost always=1
- If Q4 doesn't apply to you, mark a cross in the Q4 box and calculate the total score based on the total number of questions minus one
- From Q6.1. to Q9.4: None of the time=5, all of the time=1
- From Q14.1. to Q14.7: Not true=5, Very True=1
- Q14.8: Very true=5, Not true=1

***NB:*** *To include a section/domain in the final score, a minimum of 80% completion of items within that section is required. This is in line with the gold standard for patient-reported outcome compliance.*

**Scoring sheet**

**Section 1. General health**

| **Score** |  |  |  |  |  |  |  |  |  |  |  |
| --- | --- | --- | --- | --- | --- | --- | --- | --- | --- | --- | --- |
| **Question** | **Q2.1.** | **Q2.2.** | **Q2.3.** | **Q2.4.** | **Q3.1.** | **Q3.2.** | **Q3.3.** | **Q4.** | **Q5.1.** | **Q5.2.** | **Q5.3.** |

**Section 2. Pain**

| **Score** |  |  |  |  |  |  |  |  |
| --- | --- | --- | --- | --- | --- | --- | --- | --- |
| **Question** | **Q6.1.** | **Q6.2.** | **Q6.3.** | **Q6.4.** | **Q7.1.** | **Q7.2.** | **Q7.3.** | **Q7.4.** |

| **Score** |  |  |  |  |  |  |  |  |
| --- | --- | --- | --- | --- | --- | --- | --- | --- |
| **Question** | **Q8.1.** | **Q8.2.** | **Q8.3.** | **Q8.4** | **Q9.1.** | **Q9.2.** | **Q9.3.** | **Q9.4.** |

**Section 3. Function/activity**

| **Score** |  |  |  |  |  |  |  |  |  |  |  |
| --- | --- | --- | --- | --- | --- | --- | --- | --- | --- | --- | --- |
| **Question** | **Q11.1.** | **Q11.2.** | **Q11.3.** | **Q11.4.** | **Q11.5.** | **Q11.6.** | **Q11.7.** | **Q11.8** | **Q11.9** | **Q11.10.** | **Q11.11.** |

| **Score** |  |  |  |  |  |  |  |  |  |  |  |
| --- | --- | --- | --- | --- | --- | --- | --- | --- | --- | --- | --- |
| **Question** | **Q11.12.** | **Q11.13.** | **Q11.14.** | **Q11.15.** | **Q11.16.** | **Q11.17.** | **Q11.18.** | **Q11.19.** | **Q11.20.** | **Q11.21.** | **Q11.22.** |

**Section 4. Self-image/appearance**

**Q12.**

| **n. 1** | **n. 2** | **n. 3** | **n. 4** | **n. 5** | **n. 6** | **n. 7** | **n. 8** | **n. 9** |
| --- | --- | --- | --- | --- | --- | --- | --- | --- |


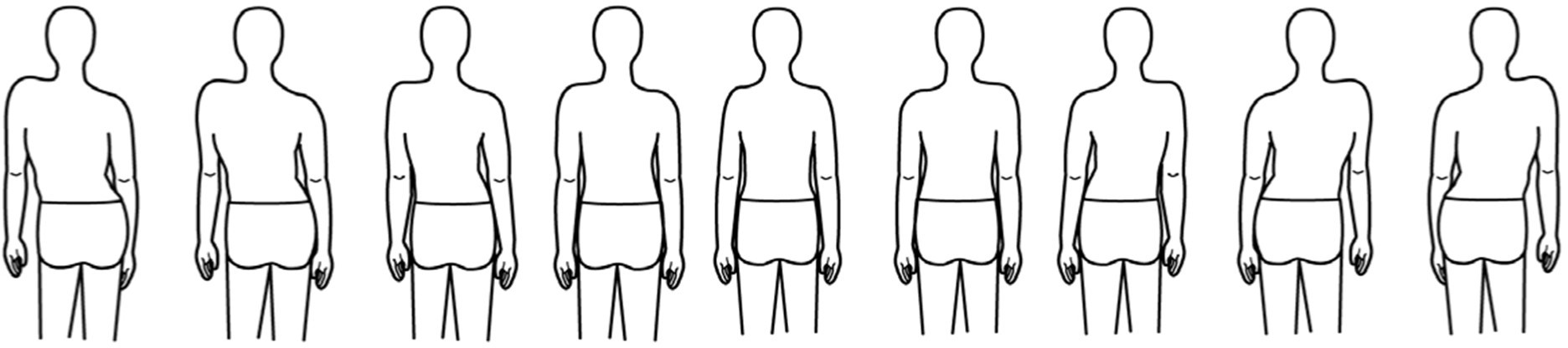


| **Score** | **1** | **2** | **3** | **4** | **5** | **4** | **3** | **2** | **1** |
| --- | --- | --- | --- | --- | --- | --- | --- | --- | --- |

| **Score** |  |  |  |  |  |  |  |  |  |  |
| --- | --- | --- | --- | --- | --- | --- | --- | --- | --- | --- |
| **Question** | **Q13.** | **Q14.** | **Q14.1.** | **Q14.2.** | **Q14.3.** | **Q14.4.** | **Q14.5.** | **Q14.6.** | **Q14.7.** | **Q14.8** |

**Section 5. Mental health**

| **Score** |  |  |  |  |  |  |  |
| --- | --- | --- | --- | --- | --- | --- | --- |
| **Question** | **Q15.1.** | **Q15.2.** | **Q15.3.** | **Q15.4.** | **Q15.5.** | **Q15.6.** | **Q15.8.** |

**Summary score**

|  | **Sum of responses** | **Questions answered (possible)** | **Mean score**  **(Sum of responses ÷ questions answered)** |
| --- | --- | --- | --- |
| **General health** |  | **(11)** |  |
| **Pain** |  | **(16)** |  |
| **Function/activity** |  | **(22)** |  |
| **Self-image/appearance** |  | **(10)** |  |
| **Mental health** |  | **(8)** |  |
| **Total** |  | **(67)** |  |
